# Diphlorethohydroxycarmalol Isolated from *Ishige okamurae* Represses High Glucose-Induced Angiogenesis In Vitro and In Vivo

**DOI:** 10.3390/md16100375

**Published:** 2018-10-10

**Authors:** K. H. N. Fernando, Hye-Won Yang, Yunfei Jiang, You-Jin Jeon, BoMi Ryu

**Affiliations:** Department of Marine Life Science, Jeju National University, Jeju 63243, Korea; hiruninfdo@gmail.com (K.H.N.F.); koty221@naver.com (H.-W.Y.); jiangyunfei0310@gmail.com (Y.J.)

**Keywords:** diphlorethohydroxycarmalol, *Ishige okamurae*, angiogenesis, diabetes, EA.hy926, zebrafish, VEGFR-2

## Abstract

Diabetes mellitus causes abnormalities of angiogenesis leading to vascular dysfunction and serious pathologies. Diphlorethohydroxycarmalol (DPHC), which is isolated from *Ishige okamurae*, is well known for its bioactivities, including antihyperglycemic and protective functions against diabetes-related pathologies. In the present study, the inhibitory effect of DPHC on high glucose-induced angiogenesis was investigated on the human vascular endothelial cell line EA.hy926. DPHC inhibited the cell proliferation, cell migration, and tube formation in cells exposed to 30 mM of glucose to induce angiogenesis. Furthermore, the effect of DPHC against high glucose-induced angiogenesis was evaluated in zebrafish embryos. The treatment of embryos with DPHC suppressed high glucose-induced dilation in the retinal vessel diameter and vessel formation. Moreover, DPHC could inhibit high glucose-induced vascular endothelial growth factor receptor 2 (VEGFR-2) expression and its downstream signaling cascade. Overall, these findings suggest that DPHC is actively involved in the suppression of high glucose-induced angiogenesis. Hence, DPHC is a potential agent for the development of therapeutics against angiogenesis induced by diabetes.

## 1. Introduction

Angiogenesis is the formation of new capillary blood vessels from existing blood vessels, and is distinct from the normal vasculogenesis of blood vessels’ formation from precursor cells [1]. Apart from its role in pathologies, angiogenesis is a vitally important process in normal growth including reproduction, development, and repair [2]. In diabetes, a high plasma glucose level causes abnormalities of angiogenesis resulting in vascular dysfunction and serious pathologies [3]. From the vascular point of view, diabetes is known as a “paradoxal disease” due to its dual role in excessive angiogenesis and insufficient angiogenesis in different organs [4]. Enhanced angiogenesis causes diabetic retinopathy [5] and nephropathy [6]. 

Although abnormal vascular growth is alleviated with antiangiogenic agents, they cause unexpected side effects including endothelial dysfunction and vessel pruning [7]. Therefore, there is a clear need for safer and more effective approaches. Recently, the screening of angiogenesis inhibitors from phytonutrients and dietary bioactives has taken much attention due to their low cost and relative safety [8]. Indeed, it was shown that marine algae-derived fucoxanthin [9] and bromophenols [8] serve as angiogenesis inhibitors.

**Figure 1:**
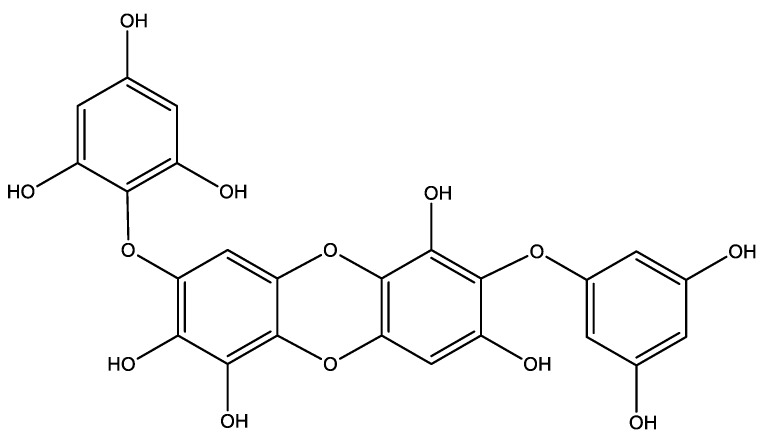
Structure of diphlorethohydroxycarmalol (DPHC), isolated from *Ishige okamurae*.

Diphlorethohydroxycarmalol (DPHC, Figure 1) is a phlorotannin that is isolated from *Ishige okamure*; it is an edible brown alga grown in the subtidal regions of Jeju island, South Korea [10]. This compound has been studied for its antidiabetic activity and protective functions against diabetic-related pathologies [11]. The protective effect of DPHC against β cell dysfunction, which is caused by the oxidative stress that is associated with diabetes, has been previously shown [12]. However, no research has been carried out to assess the protective effect of DPHC against angiogenesis resulting from high glucose levels. Therefore, in the present study, we evaluated the antiangiogenesis activity of DPHC against high glucose-induced angiogenesis in vitro and in vivo models. 

Vascular endothelial cells are treated with high concentrations of glucose in order to mimic conditions of high glucose-induced angiogenesis in vitro [13]. Hence, the vascular endothelial cell EA.hy926 was used in the present study to investigate the inhibitory effects of DPHC against high glucose-induced angiogenesis. EA.hy926 is a hybrid cell obtained by the fusion of human umbilical vein endothelial cell (HUVEC) with the human lung carcinoma cell line A549. EA.hy926 cells show characteristics of the human vascular endothelium, and its functional stability through passages, which makes it an ideal cell line for angiogenesis-related studies [14,15]. Even though the angiogenesis assays generally utilize the HUVEC isolated from the human umbilical vein, this cell shows several constraints, including limited replication potential and distinct characteristics of primary source material [16]. Therefore, compared to HUVEC, EA.hy926 cells are known to be a homogeneous experimental model assuring the reproducibility of the data [17]. 

The anti-angiogenesis activity of DPHC in EA.hy926 cells was investigated by assessing cell proliferation, migration, and tube formation, which are known as key steps in angiogenesis. The inhibition of vascular endothelial growth factor receptor 2 (VEGFR-2) is a major approach in antiangiogenic drug development [18]. The expression levels of VEGFR-2 and several downstream signaling molecules were examined to elucidate the mechanism of action of DPHC against high glucose-induced angiogenesis. Furthermore, the effect of DPHC against high glucose-induced angiogenesis was evaluated in vivo model using zebrafish embryos.

## 2. Results

### 2.1. Glucose-Induced Angiogenesis in EA.hy926 Cells

**Figure 2:**
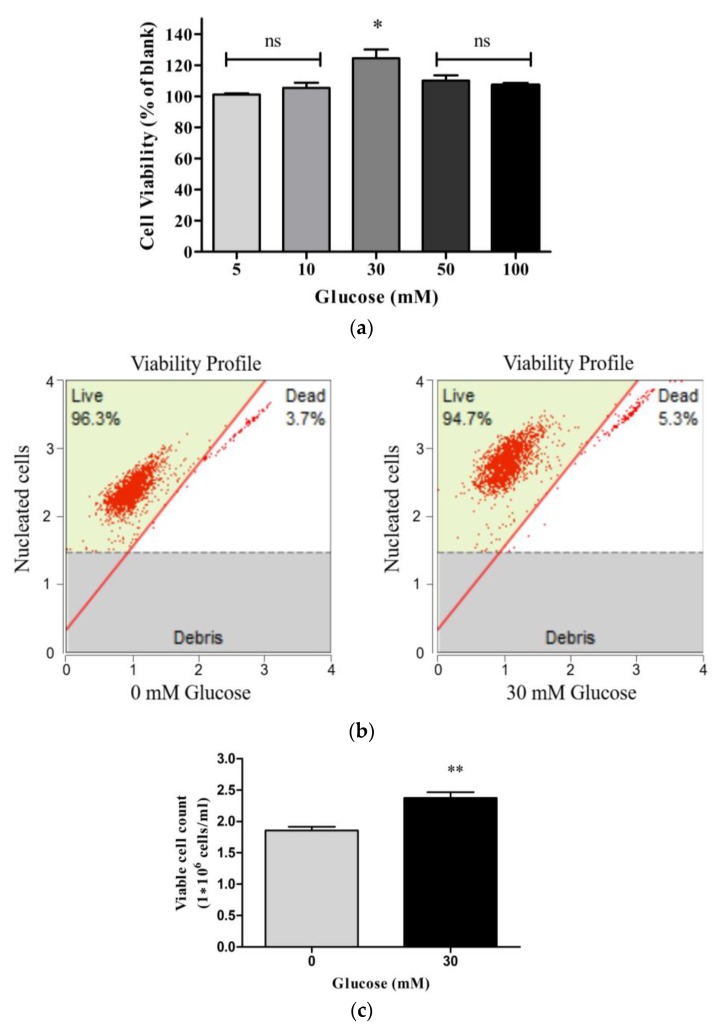
Effect of glucose on the proliferation of vascular endothelial cell EA.hy926. (**a**) Cell viability upon treatment with glucose for 24 h. Cells were incubated with increasing concentrations of glucose (zero mM, five mM, 10 mM, 30 mM, 50 mM, and 100 mM) for 24 h, and cell viability was determined by the MTT assay. Results are normalized to blank (0 mM of glucose). Cell viability upon the treatment with 30 mM of glucose for 24 h was assessed using the Muse™ Count and Viability Kit; representative viability profiles (dot plots) (**b**) and viable cell count are shown (**c**). The results were obtained using Muse™ Cell Analyzer. Results are shown as means ± SD of three independent experiments; ns, not significant, * *p* ˂ 0.05, ** *p* ˂ 0.01 compared with the blank (0 mM of glucose).

Prior to assessing the antiangiogenic effects of DPHC in vitro, EA.hy926 cells were treated with different concentrations of glucose (zero mM, five mM, 10 mM, 30 mM, 50 mM, and 100 mM) to confirm the concentration that could induce the angiogenesis condition in EA.hy926 cells. According to the MTT (3-[4,5-dimethylthiazol-2-yl]-2,5 diphenyltetrazolium bromide) assay results (Figure 2a) the endothelial cell viability was significantly increased at 30 mM glucose concentration. After 24 h, EA.hy926 cells showed 124.8 ± 5.5% of cell viability compared to the blank (no glucose treatment). In order to further confirm the effect of 30 mM of glucose in the cell proliferation, the viable cell count was analyzed using the Muse™ Cell Analyzer, and the results showed a significant cell proliferation at 30 mM of glucose concentration, compared to the blank (Figure 2c). These results suggested that 30 mM of glucose could be used for the in vitro angiogenesis induction in EA.hy926 cells, and therefore, this concentration was used for further studies.

### 2.2. Cytotoxicity of DPHC in EA.hy926 Cells with and without Glucose Treatment

**Figure 3:**
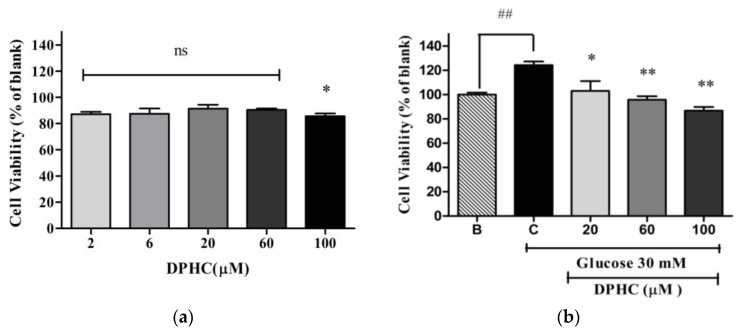
Effect of diphlorethohydroxycarmalol (DPHC) on the proliferation of EA.hy926 cells. (**a**) Cytotoxicity of DPHC in EA.hy926 cells. Cells were incubated with different concentrations of DPHC (zero μM, two μM, six μM, 20 μM, 60 μM, and 100 μM) for 24 h, and cell viability was determined by MTT assay. Results are normalized to blank (0 μM DPHC). (**b**) The anti-proliferation effect of DPHC in high glucose-treated EA.hy926 cells. Cells were treated without glucose or DPHC (B, blank), with 30 mM of glucose without DPHC (C, control) and with different concentrations of DPHC (20 μM, 60 μM, and 100 μM) together with 30 mM of glucose. Cells were incubated for 24 h and cell viability was measured by MTT assay. Effect of 30 mM of glucose on cell proliferation is compared with B; blank (0 mM glucose + 0 μM DPHC), ^##^ *p* ˂ 0.01. Anti-proliferation effect of DPHC in high glucose-treated cells is normalized to C; control (30 mM glucose + 0 μM DPHC). The data are shown as means ± SD of three independent experiments; ns, not significant * *p* ˂ 0.05, ** *p* ˂ 0.01.

The MTT method was used to determine the cytotoxicity of DPHC in EA.hy926 cells. Cells were treated with two μM, six μM, 20 μM, 60 μM, and 100 μM of DPHC concentrations for 24 h. As shown in Figure 3a, cell viability was found to be 87.1 ± 1.8%, 88.5 ± 4.1%, 91.3 ± 2.9%, 90.4 ± 1.1% and 85.6 ± 2.1% with DPHC concentrations of two μM, six μM, 20 μM, 60 μM, and 100 μM respectively, normalized to the blank. Based on these results, 20 μM, 60 μM, and 100 μM of DPHC concentrations were selected to assess the antiangiogenic effect of DPHC. Cell proliferation is regarded as one of the initial steps in angiogenesis [19]. To evaluate whether DPHC inhibits high glucose-induced proliferation, MTT assay was performed. As shown in Figure 3b, treatment with 30 mM of glucose increased cell viability significantly (124.3 ± 3.0%) compared to the blank. The results show that high glucose-induced cell proliferation decreased significantly in a concentration-dependent manner with DPHC treatment. Cell viability was found to be 103.2 ± 8.1%, 95.8 ± 2.8% and 86.8 ± 2.9% with DPHC concentrations of two μM, six μM, 20 μM, 60 μM, and 100 μM, respectively, in high glucose-treated cells. These results revealed that DPHC repressed high glucose-induced cell proliferation.

### 2.3. DPHC Inhibited High-Glucose Induced Cell Migration

**Figure 4:**
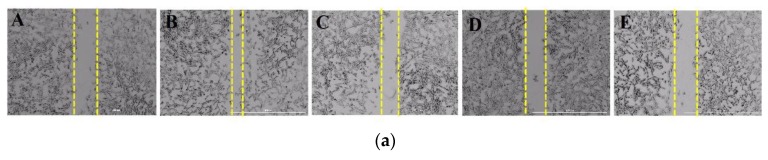

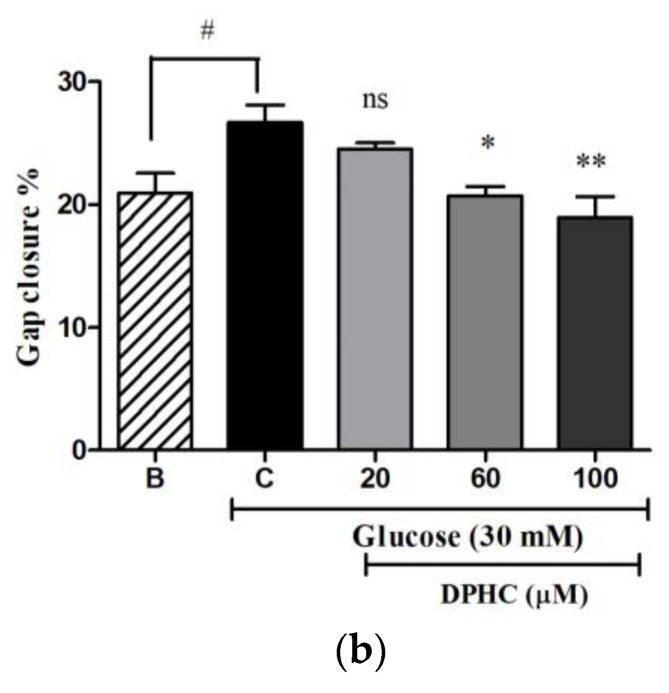
(**a**) DPHC inhibited the migration of EA.hy926 cells treated with high glucose concentrations. Cells were treated with glucose (30 mM) together with DPHC (20 μM, 60 μM, and 100 μM), blank (0 mM glucose + 0 μM DPHC) and control (30 mM glucose + 0 μM DPHC). A scratch was made in the middle of the well and the initial gap length (0 h) and the final gap length (after 12 h of incubation) were photographed and gap closure percentage was determined. A: 0 mM glucose + 0 μM DPHC; B: 30 mM glucose + 0 μM DPHC; C: 30 mM glucose + 20 μM DPHC; D: 30 mM glucose + 60 μM DPHC; E: 30 mM glucose + 100 μM DPHC. (**b**) Quantitative evaluation of migration inhibition of DPHC in high glucose-induced EA.hy926 cells. Effect of 30 mM of glucose is compared with B; blank (0 mM glucose + 0 μM DPHC), ^#^ *p* ˂ 0.05. Percentage gap closure is normalized to C: control (30 mM glucose + 0 μM DPHC); ns, not significant, * *p* ˂ 0.05, ** *p* ˂ 0.01.

Endothelial cell migration is one of the key steps in angiogenesis [8]. To determine the influence of DPHC on the migration of EA.hy926 cells, gap closure assay was employed (Figure 4a,b). Cell migration was expressed as a percentage of gap closure. Increased gap closure percentage is an indicative of higher cell migration. The results showed that treatment with 30 mM of glucose significantly increased the gap closure percentage (26.67 ± 1.9%), while DPHC could significantly reduce the high glucose-induced gap closure percentage in cells treated with glucose in a concentration-dependent manner. In fact, gap closure percentage was reduced to 23.83 ± 0.6%, 20.72 ± 1.1%, and 18.9 ± 1.9% with DPHC at concentrations of 20 μM, 60 μM, and 100 μM, respectively. Given that gap closure is directly related to cell migration, these results suggested that DPHC inhibits the migration of EA.hy926 cells, thus contributing to its antiangiogenic effect.

### 2.4. DPHC Abrogated the Capillary-Like Structure Formation of High-Glucose Treated EA.hy 926 Cells

**Figure 5:**
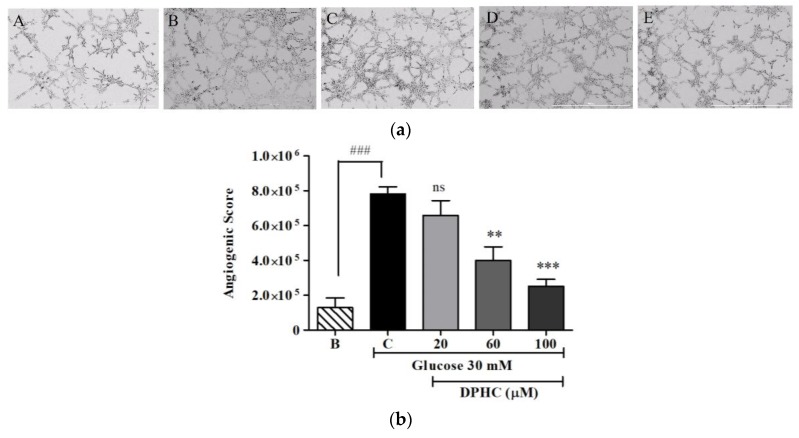
(**a**) DPHC abrogated the high glucose-induced formation of capillary-like structures in Matrigel. Cells were seeded in Matrigel without glucose treatment (B, blank), or with 30 mM of glucose treatment (C, control) and with 30 mM of glucose together with DPHC at 20 μM, 60 μM, and 100 μM. Cells were incubated for 6 h and observed using a phase-contrast microscope (4×). A: 0 mM glucose + 0 μM DPHC; B: 30 mM glucose + 0 μM DPHC; C: 30 mM glucose + 20 μM DPHC; D: 30 mM glucose + 60 μM DPHC; E: 30 mM glucose + 100 μM DPHC. (**b**) Quantified angiogenic score. The effect of 30 mM of glucose is compared with B, the blank (0 mM glucose + 0 μM DPHC); ^###^ *p* ˂ 0.001. Effect of DPHC on high glucose-induced capillary formation is normalized to C: control (30 mM glucose + 0 μM DPHC); ns, not significant, ** *p* ˂ 0.01, *** *p* ˂ 0.001.

The formation of capillary-like structures in vascular endothelial cells is the final and important step in angiogenesis [19]. Tube formation/capillary-like structure formation was assessed by growing the cells on Matrigel (Figure 5a,b). Angiogenic score was determined for quantitative analysis. The capillary-like tube structure network formation was significantly enhanced with 30 mM of glucose treatment, and showed an angiogenic score of 10^5^(7.28 ± 0.16). Cells were treated together with 30 mM of glucose and different concentrations (20 μM, 60 μM, and 100 μM) of DPHC in order to observe the effect of DPHC on high glucose-induced capillary formation. The results showed that the angiogenic score was significantly decreased to 10^5^(6.59 ± 0.27), 10^5^(4.33 ± 0.21), and 10^5^(2.5 ± 0.18) with DPHC treatments of 20 μM, 60 μM, and 100 μM, respectively. These results suggested that DPHC inhibited the capillary-like structure formation in high glucose-induced cells.

### 2.5. DPHC Interfered with VEGFR-2 and Downstream Signaling Molecules

**Figure 6:**
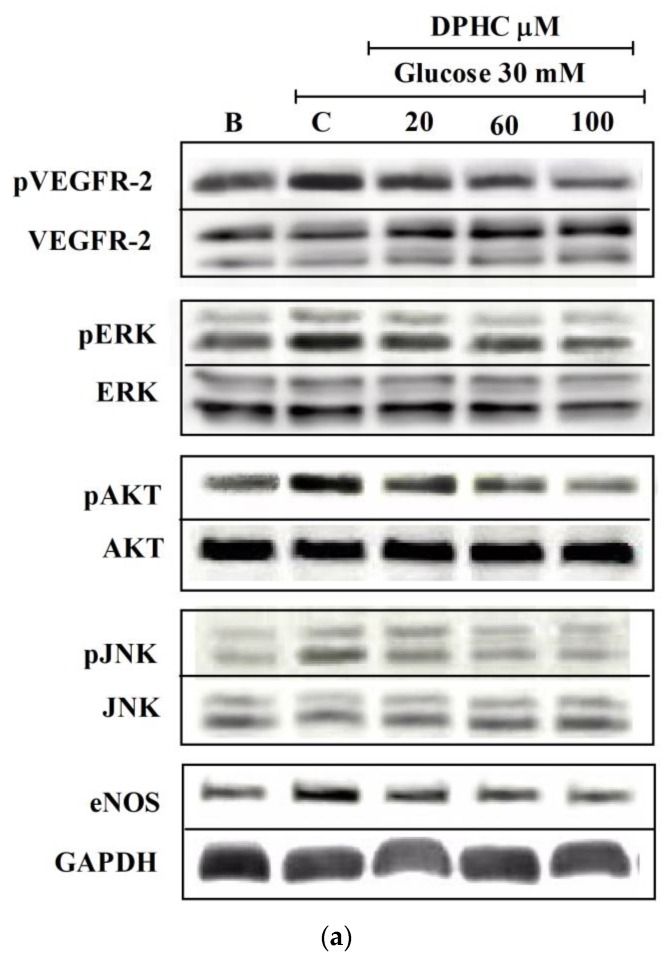

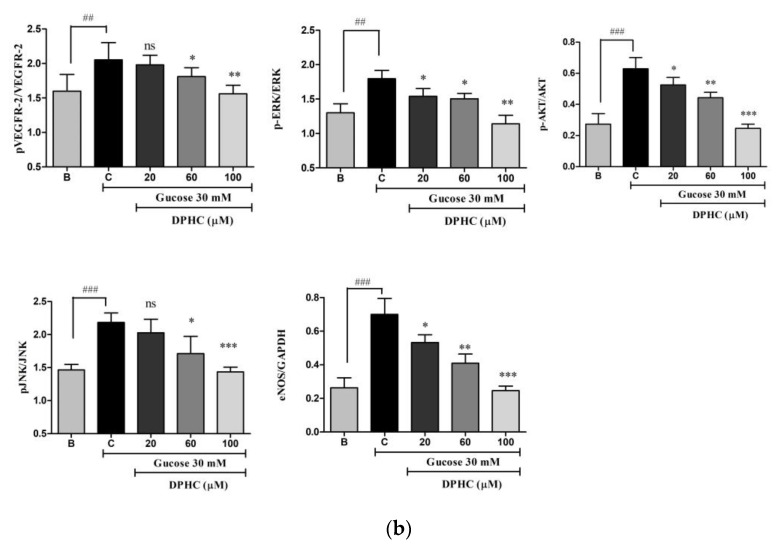
DPHC interfered with vascular endothelial growth factor receptor 2 (VEGFR-2) and downstream signaling molecules. The effect of DPHC on high glucose-induced phosphorylation of VEGFR-2 and downstream signaling molecules including phospho-extracellular signal-regulated kinase (pERK)/extracellular signal-regulated kinase (ERK), phospho-protein kinase B (pAKT)/ protein kinase B (AKT), phospho-c-Jun N-terminal kinase (pJNK)/c-Jun N-terminal kinase (JNK), and endothelial nitric oxide synthase (eNOS) were detected by Western blotting (**a**). Protein expression and phosphorylation were respectively normalized to glyceraldehyde-3-phosphate-dehydrogenase GAPDH or respective total protein expression by image J software (**b**). Effect of 30 mM of glucose is compared with B, the blank. Effect of DPHC is normalized to C; control (30 mM glucose + 0 μM DPHC), ns; not significant, * *p* ˂ 0.05, ** *p* ˂ 0.01, *** *p* ˂ 0.001.

To determine whether the antiangiogenic effect of DPHC is associated with VEGFR-2 and downstream signaling molecules such as protein kinase B (AKT), extracellular signal-regulated kinase (ERK), c-Jun N-terminal kinase (JNK), and endothelial nitric oxide synthase (eNOS), we used Western blotting. As shown in Figure 6, glucose treatment can significantly induce phosphorylated VEGFR-2 (pVEGFR-2) expression compared to the blank. The treatment of cells with DPHC results in the reduction of high glucose-induced pVEGFR-2 expression. As shown in Figure 6, DPHC could downregulate not only the VEGFR-2 expression but also some of the important downstream molecules, including phospho-AKT (pAKT), phospho-ERK (pERK), phospho-JNK (pJNK), and eNOS.

### 2.6. DPHC Inhibited High Glucose-Induced Dilation of Hyaloid Retinal Vessel Diameter and the Whole Body Vessel Formation in Zebrafish Embryos

**Figure 7:**
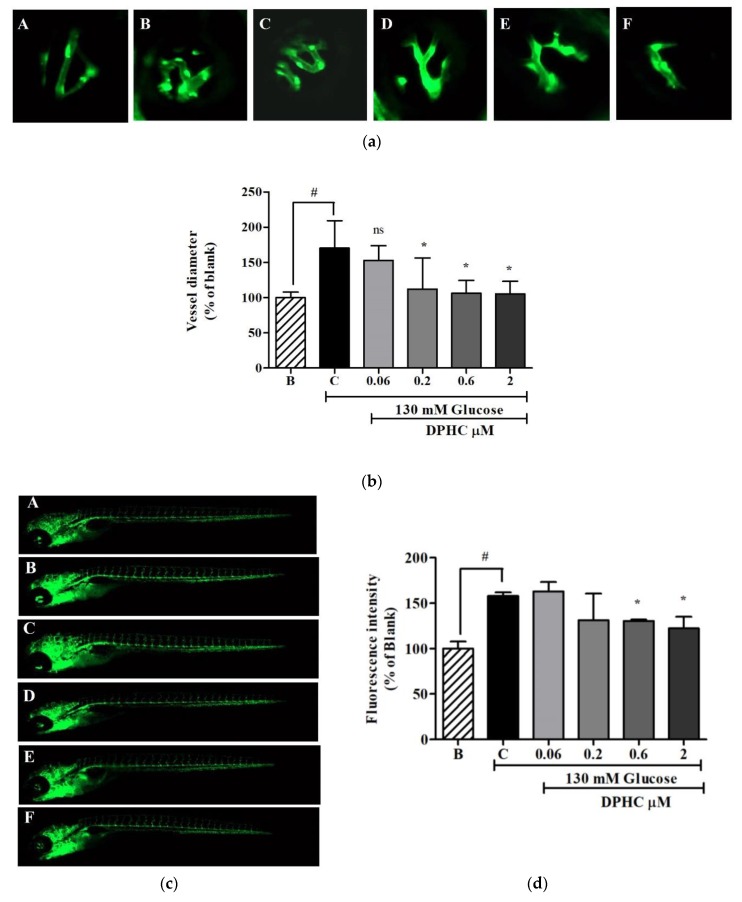
DPHC inhibited high glucose-induced dilation in hyaloid retinal vessel diameter and the whole body vessel formation in the zebrafish transgenic (*flk*:EGFP) embryo. A: 0 mM glucose + 0 μM DPHC; B: 130 mM glucose + 0 μM DPHC; C: 130 mM glucose + 0.06 μM DPHC; D: 130 mM glucose + 0.2 μM DPHC; E: 130 mM glucose + 0.6 μM DPHC; F: 130 mM glucose + 2 μM DPHC. (**a**) Images of hyaloid retinal vessels of the zebrafish transgenic (*flk*:EGFP) embryo taken with florescence microscopy (10×). (**b**) Quantification of the hyaloid retinal vessel diameter. (**c**) Images of the zebrafish transgenic (*flk*:EGFP) embryo’s whole body taken with florescence microscopy (4×). (**d**) Quantification of the whole body florescence intensity. The effect of 130 mM of glucose is compared with the blank (130 mM glucose + 0 μM DPHC), ^#^ *p* ˂ 0.05. The effect of DPHC against high glucose-induced vessel growth is normalized to the control (130 mM glucose + 0 μM DPHC); ns, not significant, * *p* ˂ 0.05.

To further confirm the antiangiogenic activity of DPHC in vivo, the vessel formation of the zebrafish transgenic (*flk*:EGFP) embryo was examined. Zebrafish is widely used in angiogenesis studies. Jung et al. [20] suggested that 130 mM of glucose is a standard treatment to induce changes in the retinal vessels. In the present study, the zebrafish transgenic (*flk*:EGFP) embryo was treated with 130 mM of glucose to induce angiogenesis in retinal vessels and the whole body. As shown in Figure 7a, hyaloid retinal vessel diameter is significantly increased (170.1 ± 15.25% compared to blank) upon treatment with 130 mM of glucose. Treatment with 0.06 μM, 0.2 μM, 0.6 μM, and 2 μM of DPHC resulted in a decrease in hyaloid retinal vessel diameter to 152.0 ± 9.4%, 112.3 ± 16.56%, 106.2 ± 7.5%, and 105.2 ± 6.8%, respectively (Figure 7b). Exposure to DPHC also inhibited the high glucose-induced vessel formation in the whole body (Figure 7c). The florescence intensity of the whole body treated with glucose increased to 157.9 ± 2.92%. DPHC at 0.06 μM, 0.2 μM, 0.6 μM, and 2 μM reduced the high glucose-induced vessel formation by 163.2 ± 7.23%, 131.4 ± 18.56%, 130.3 ± 1.15%, and 122.3 ± 7.5%, respectively (Figure 7d). These results showed the antiangiogenesis effect of DPHC against high glucose-induced angiogenesis in vivo.

## 3. Discussion

Phlorotannins are isolated from marine brown algae, and are well known for their potential as a functional ingredient in food products, pharmaceuticals, and cosmeceuticals [21]. DPHC is a phlorotannin that is isolated from the marine brown alga *Ishige okamurae.* DPHC has been reported to show several biological activities, including antiviral [10], antioxidant [22], antiinflammatory [23], and antihyperglycemic activities [11]. Diabetes mellitus type II is characterized by several metabolic changes, including insulin resistance, hyperinsulinemia, and hyperglycemia, which lead to vascular changes [24] and result in enhanced angiogenesis by the degradation of the extracellular matrix, and increased proliferation, survival, and migration, as well as morphological changes of endothelial cells and the development of the vascular network [25]. In the present study, the inhibitory activity of DPHC against the endothelial changes that are induced by a high concentration of glucose in in vitro and in vivo models was evaluated.

As an initial approach, vascular endothelial cells EA.hy926 were treated with different concentrations of glucose to mimic the angiogenesis conditions in vitro. According to the results, 30 mM of glucose treatment showed a significant increase in cell viability compared to the blank. During the angiogenesis, endothelial cells undergo multiple independent processes, including detachment from basement membranes, proliferation, migration, and maturation [26]. 

EA.hy926 cells treated with 30 mM of glucose showed a significant induction in cell proliferation, cell migration, and capillary structure formation. DPHC showed, in a concentration-dependent manner, antiangiogenesis activity by the significant inhibition of high glucose-induced cell proliferation, migration, and capillary structure formation. An antiangiogenesis effect through the inhibition of essential steps of angiogenesis has been previously reported for other antiangiogenesis agents such as pseudolaric acid B [27].

*In vitro* findings were further supported by in vivo studies using the transgenic zebrafish (*flk*:EGFP) embryo. Zebrafish has emerged as a new animal model that is suitable for studying angiogenesis inhibitors targeting the eye, as well as the whole body [28]. Specifically, zebrafish embryos exposed to high concentrations of glucose show the dilation of the hyaloid retinal vessel and variations of the tight junction proteins, providing a platform to study diabetic retinopathy [20]. In this study, DPHC could significantly inhibit the high glucose-induced dilation in retinal vessel diameter in the zebrafish embryo. High glucose-induced retinal vessel diameter was rescued upon the treatment with DPHC. The similar effect has been reported in zebrafish by Wu et al. [29] in CoCl_2_-induced retinal angiogenesis after the treatment with anti-VEGF. Moreover, DPHC was assessed for its activity against vessel formation in the whole body. The measured fluorescence intensities indicated that treatment with DPHC reversed the vessel formation significantly against the high glucose-induced angiogenesis. The in vivo results confirmed that DPHC can serve as an antiangiogenic agent for diabetic-related angiogenesis. 

Most of the physiological and pathological responses of endothelial cells, including cell proliferation, migration, and survival are triggered by VEGFR-2 [30]. The treatment of high glucose showed that the increased phosphorylation of VEGFR-2 in the vascular endothelial cells and DPHC treatment suppressed the phosphorylation of VEGFR-2. The receptor acts as the principal mediator of downstream signals of angiogenesis, including eNOS, AKT, JNK, and ERK [31]. ERK plays an important role in endothelial cell proliferation [32], while JNK activates cell proliferation and migration [33]. The phosphorylation of AKT activates endothelial survival [34]. eNOs is known to be an important key molecule in angiogenesis, which promotes vascular permeability [35]. Our results showed that DPHC decreased the phosphorylation of VEGFR-2, ERK, AKT, and JNK, and the expression of eNOS. These results supported that DPHC treatment suppressed the VEGFR-2 and the downstream signaling cascade simultaneously. Taken together, this study revealed that DPHC is actively involved in suppressing high glucose-induced angiogenesis in vitro and in vivo models. 

## 4. Materials and Methods 

### 4.1. Materials

DPHC was isolated from I. okamurae according to the method used in our previous study [23] with a slight modification. Briefly, I. okamurae was collected from Seongsan in Jeju Island, South Korea in April 2016. Dried alga (500 g) was extracted with 50% (*v*/*v*) aqueous ethanol (5 L) under stirring at room temperature (RT) for 24 h. The extract was concentrated and freeze-dried (87 g) until needed. The extract (500 mg) was fractionated using centrifugal partition chromatography (CPC 240, Tokyo, Japan). The two-phase solvent system was composed of n-Hexane:EtOAC:MeOH:H_2_O (1:9:4.5:6.5, *v*/*v*). DPHC with 97% purity was isolated by semi-preparative HPLC column (YMC-Pack ODS-A,10 × 250 mm, 5 μm), eluting with an isocratic system of 32% CH_3_CN containing 0.1% formic acid (Sigma-Aldrich 229 at a flow rate of two mL/min, St. Louis, MO, USA) (Agilent, 1260 Infinity II gradient LC system VL, Palo Alta, CA, USA). The identity of DPHC (Figure 1) was verified by MS fragmentation of m/z 512 at an ultrahigh resolution Q-TOF LC-MS/MS coupled with an electrospray ionization (ESI) resource (Figure S1, Supplementary materials) (maXis-HD, Bruker Daltonics, Breman, Germany). Mass spectra were acquired in a positive ion mode by a scanning m/z range from 50 to 2000. The ^1^H and ^13^C spectra of DPHC (Figure S2, Supplementary materials) were recorded in DMSO-d6 on a Bruker Biospin Advance II 800 NMR spectrometer at the Korea Basic Science Institute (KBSI) in Ochang, South Korea.

### 4.2. Cell Line Culture

Endothelial EA.hy926 cells (American Type Culture Collection, Rockville, MD, USA) were cultured in Dulbecco’s modified Eagle’s medium (DMEM; Gibco, Life Technologies, New York, NY, USA) supplemented with 10% fetal bovine serum (FBS; Merck, Sacramento, CA, USA), 1% penicillin, and a streptomycin mixture, and incubated at 37 °C in a humidified atmosphere containing 5% CO_2_. Cells plates were split 1:3 when they reached 70–80% confluence. Passage numbers between four and 10 were used for all of the experiments.

### 4.3. Cell Treatment with Glucose

EA.hy926 cells were plated onto a 96-well plate with three replicates for each concentration. Following 24 h of incubation at 37 °C, different concentrations (0 mM, 5 mM, 10 mM, 30 mM, 50 mM, and 100 mM) of D-glucose (Sigma-Aldrich) dissolved in serum-free DMEM were added to each well and incubated for 24 h. Cell viability was estimated via a colorimetric MTT method [36]. Subsequently, the medium was removed and treated with 50 μL of MTT reagent (2 mg/mL in phosphate-buffered saline [PBS]) for 4 h. Following a removal of MTT, purple crystals were dissolved in 100 μL of DMSO (Amresco, Life Sciences, Solon, OH, USA), and the absorbance was measured at 540 nm using a micro plate reader (Synergy HT, BioTek Instruments, Winooski, VT, USA). Cell viability at different glucose concentrations was expressed as a percentage of the blank (0 mM of glucose).

### 4.4. Analysis of Cell Proliferation by Flow Cytometry

The cell proliferation was further studied at a glucose concentration of 30 mM. EA.hy926 cells were seeded in six-well plates at a density of 1 × 10^6^ and incubated overnight. Cells were treated with 0 mM and 30 mM of glucose and incubated. After 24 h, the cells were harvested by trypsinization, and then the cells were washed with ice-cold phosphate-buffered saline (PBS) and incubated with Muse™ cell count and viability reagent (Milipore, Manufacturer, Billerica, MA, USA) for five minutes. The cell viability count was measured by Muse™ Cell Analyzer (Milpore, Hayward, CA, USA). 

### 4.5. Determination of Cytotoxicity of DPHC in EA.hy926 Cells

EA.hy926 cells (1 × 10^5^ cells/well) were seeded in a 96-well plate and incubated for 24 h. The cells were treated with various concentrations of DPHC (0 μM, 2 μM, 6 μM, 20 μM, 60 μM, and 100 μM) and further incubated for 24 h. Subsequently, cell viability was determined using the MTT assay as described above (2.3), and was expressed as a percentage of the blank (0 μM DPHC).

### 4.6. Determination of Anti-Proliferative Activity of DPHC against High Glucose-Treated EA.hy926 Cells

To assess whether DPHC affects high glucose-induced cell proliferation, EA.hy926 cells were treated with glucose (30 mM) and DPHC (20 μM, 60 μM, and 100 μM) or the blank (0 mM glucose + 0 μM DPHC) and control (30 mM glucose + 0 μM DPHC). Cells were incubated for 24 h and cell viability was measured by the MTT assay as described above (Section 2.3), and expressed as a percentage of the blank. The effect of 30 mM of glucose was compared with the blank (0 mM glucose + 0 μM DPHC). The anti-proliferative effect of DPHC on high glucose-induced proliferation was compared to the control (30 mM glucose + 0 μM DPHC).

### 4.7. Scratch Wound Cell Migration Assay

EA.hy926 cells were seeded in a 96-well plate at a density of 1 × 10^5^ per well. After obtaining 80% cell confluence, a scratch was made in the middle of the well by a 10-μL tip. After scrapping, cells were washed with PBS twice, and incubated with DMEM (Dulbecco’s modified Eagle’s medium) containing glucose (30 mM) together with DPHC (10 μg/mL, 30 μg/mL, or 50 μg/mL) or the blank (0 mM glucose + 0 μM DPHC) and control (30 mM glucose + 0 μM DPHC). The initial gap width (at 0 h) and the final gap width (after 12 h) were assessed using photography, and gap lengths were quantified using the image J software at three different points and averaged. The gap closure percentage was calculated as follows:

$$\%\;\mathrm{Gap}\;\mathrm{Closure}\;=\frac{\mathrm{Initial}\;\mathrm{gap}\;\mathrm{length}-\mathrm{Final}\;\mathrm{gap}\;\mathrm{length}}{\mathrm{Initial}\;\mathrm{gap}\;\mathrm{length}}\times 100\%$$

### 4.8. Tube Formation Assay

The tube formation assay was performed using the capillary-like structure formation ability of EA.hy926 cells in Matrigel. A 96-well plate was coated with Matrigel (BD Biosciences) and allowed to polymerize by incubating at 37 °C for 30 min. Confluent EA.hy926 cells, cultured in T75 flasks, were trypsinized and diluted in DMEM. The cell suspension was divided into 15-mL Falcon tubes containing an equal number of cells (3 × 10^3^) and centrifuged. Cell pellets were suspended in different solutions including the blank (0 mM glucose + 0 μM DPHC), control (30 mM glucose + 0 μM DPHC), and different concentrations of DPHC (10 μM, 30 μM, and 50 μM) together with 30 mM of glucose. Cells were then seeded on Matrigel-coated wells and incubated for six hours. Cultures were photographed using 4× magnification, and all of the results were quantified using the plug-in “Angiogenesis Analyzer” from the image J. Angiogenic score was determined by using the following formula [37]:

Angiogenic score=number of branches×total branch length 

### 4.9. Western Blotting

After 24 h of incubation, cells were harvested. Cytosolic proteins and membrane proteins were extracted using a membrane protein extraction kit (Thermo Scientific). The resulting protein content was quantified by a Pierce™ BCA protein Assay Kit (Thermo Scientific). An equal amount of cytosolic proteins and membrane proteins (30 μg) were separated using 7.5% or 12% SDS-PAGE and transferred to the nitrocellulose blotting membrane (GE Healthcare Life Science, Chicago, IL, USA). Membranes were blocked with non-fat dry milk for 3 h at room temperature and incubated with primary antibodies, including phosphorylated and/or total VEGFR-2, ERK, AKT, JNK, eNOS, and GAPDH (housekeeping gene, Cell Signaling Technology, Danvers, MA, USA). After overnight incubation at 4 °C, membranes were then incubated with respective secondary antibodies conjugated to horseradish peroxidase for 2 h at room temperature and developed with chemiluminescence reagent (maximum sensitivity substrate, Thermo Scientific, Rockford, IL, USA). Immunoreactive bands were then visualized by a Fusion Solo apparatus (Vilber Lourmat, Collegien, France). The protein expressions, including phosphorylated forms, were normalized to the respective total form or GAPDH using the image J software (NIH, Bethesda, MD, USA).

### 4.10. Zebrafish Embryo Treatment with High Glucose and DPHC

Zebrafish showing high glucose-induced angiogenesis was developed by treating with glucose according to the method described by Jung et al. [20]. Briefly, the zebrafish transgenic (*flk*:EGFP) embryos were maintained in 24-well plates containing embryonic water. Embryos were treated with 130 mM of glucose at three days post-fertilization (3 dpf), Embryos were untreated or treated with different concentrations of DPHC (0.06 μM, 0.2 μM, 0.6 μM, or 2 μM) at six dpf. After six hours of treatment, embryos were photographed using fluorescence microscope (LIONHEART^FX^ automated live cell imager); 10× magnification was used to measure the width of the hyaloid retinal vessel, and 4× magnification was used to evaluate the florescence intensities in the whole body. The width of the hyaloid retinal vessel diameter was measured at five different places using Gen5 3.04 software (Bio Tek, Winooski, VT, USA) and averaged. The florescence intensities of the whole body were determined using the Image J software. The corrected total object fluorescence (CTOF) was calculated.

CTOF = Integrated Density − (Area of selected object × Mean fluorescence of background readings)

The images were represented as follows: A, 0 mM glucose + 0 μM DPHC; B, 130 mM glucose + 0 μM DPHC; C, 130 mM glucose + 0.06 μM DPHC; D, 130 mM glucose + 0.2 μM DPHC; E, 130 mM glucose + 0.6 μM DPHC; F, 130 mM glucose + 2 μM DPHC.

### 4.11. Statistical Analysis

Data analysis was performed using GraphPad Prism or Microsoft Excel. Data were expressed as mean ± SD from three independent experiments and analyzed statistically using two-way ANOVA, and significant differences between treatment means were determined using Dunnett’s multiple range tests. Significance was accepted at *p* < 0.05 levels.

## 5. Conclusions

DPHC isolated from *Ishige okamure* has been found to possess multiple biological activities. In the present study, DPHC showed antiangiogenesis activity by the inhibition of high glucose-induced cell proliferation, migration, and capillary structure formation. DPHC suppressed the high glucose-induced vessel formation in zebrafish transgenic (*flk*:EGFP) embryo. Altogether, DPHC showed both in vitro and in vivo inhibitions against high glucose-induced angiogenesis. Therefore, DPHC could have the potential to develop therapeutics against angiogenesis induced by diabetes.

## Supplementary Materials

The following are available online at http://www.mdpi.com/1660-3397/16/10/375/s1, Figure S1: Mass data and chemical structure (Inner panel) of DPHC isolated from Ishige okamurae, Figure S2: 1H (a) and 13C (b) nuclear magnetic resonance (NMR) spectra of DPHC isolated from Ishige okamurae.
