# Supplementary material for: Diphlorethohydroxycarmalol Isolated from Ishige okamurae Represses High Glucose-Induced Angiogenesis In Vitro and In Vivo
Source: Mar Drugs. 2018 Oct 10;16(10):375. doi: 10.3390/md16100375 (PMC6215322; doi:10.3390/md16100375)
Supplement: Supplementary file 1 [file marinedrugs-16-00375-s001.pdf]

Supplementary Materials

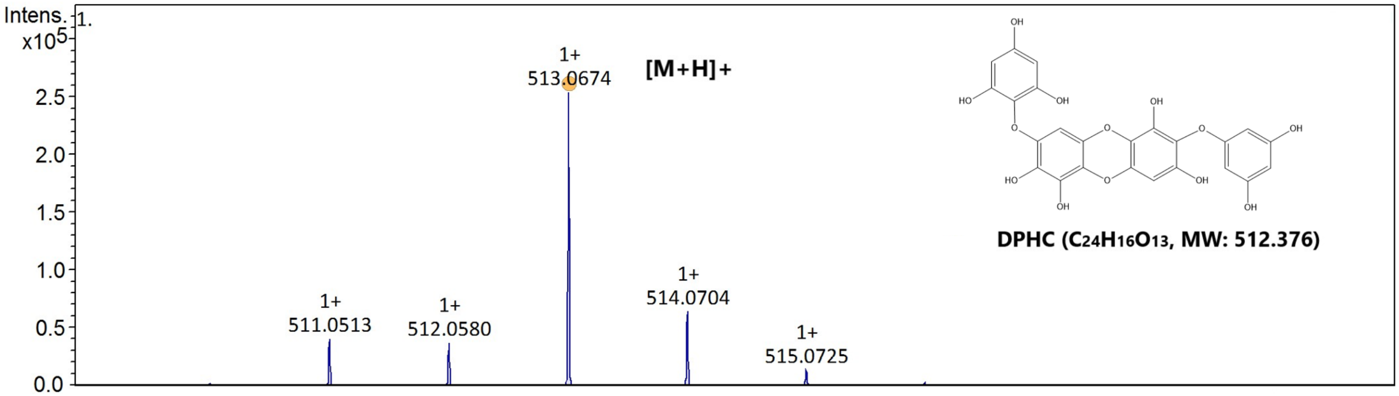

Figure S1. Mass data and chemical structure (Inner panel) of DPHC isolated from *Ishige okamurae.*

Supplementary Materials

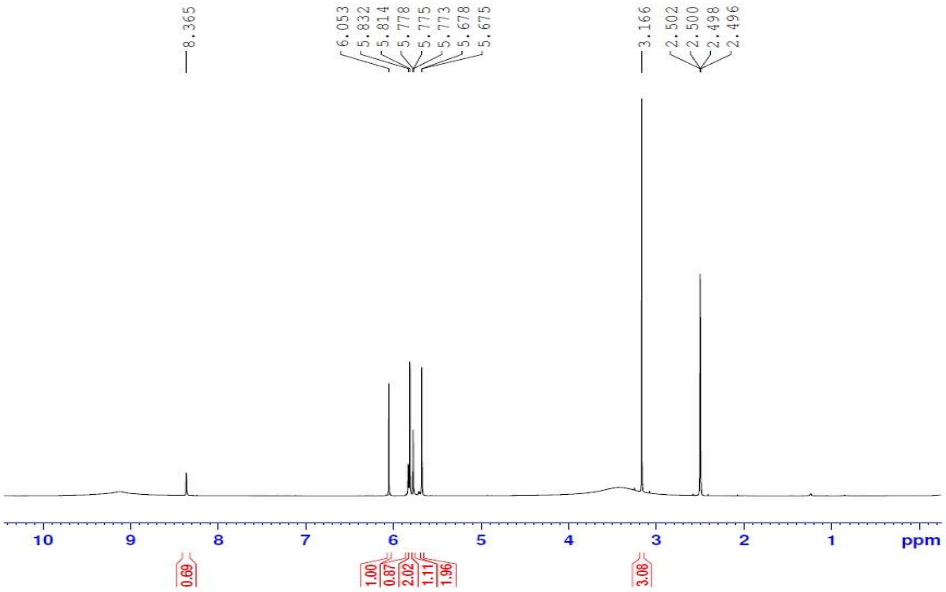

(a)

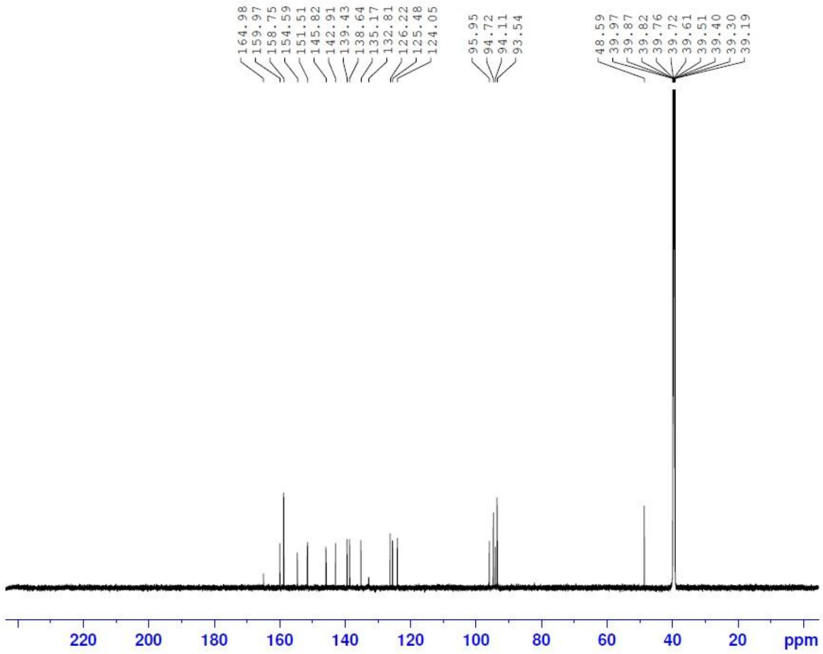

(b)

Figure S2. ^{1}H (a) and ^{13}C (b) nuclear magnetic resonance (NMR) spectra of DPHC isolated from *Ishige okamurae.*
